# Unravelling the Single-Stranded DNA Virome of the New Zealand Blackfly

**DOI:** 10.3390/v11060532

**Published:** 2019-06-08

**Authors:** Simona Kraberger, Kara Schmidlin, Rafaela S. Fontenele, Matthew Walters, Arvind Varsani

**Affiliations:** 1 The Biodesign Center for Fundamental and Applied Microbiomics, Center for Evolution and Medicine, School of Life Sciences, Arizona State University, Tempe, AZ 85287-5001, USA; simona.kraberger@asu.edu (S.K.); Kara.Schmidlin@asu.edu (K.S.); rafasfontenele@asu.edu (R.S.F.); 2 School of Biological Sciences, University of Canterbury, Christchurch 8140, New Zealand; matt.walters@canterbury.ac.nz; 3 Structural Biology Research Unit, Department of Integrative Biomedical Sciences, University of Cape Town, Rondebosch, Cape Town 7700, South Africa

**Keywords:** blackfly, sandfly, *Austrosimulium sp.*, CRESS DNA virus, *Circoviridae*, *Genomoviridae*

## Abstract

Over the last decade, arthropods have been shown to harbour a rich diversity of viruses. Through viral metagenomics a large diversity of single-stranded (ss) DNA viruses have been identified. Here we examine the ssDNA virome of the hematophagous New Zealand blackfly using viral metagenomics. Our investigation reveals a plethora of novel ssDNA viral genomes, some of which cluster in the viral families *Genomoviridae* (*n* = 9), *Circoviridae* (*n* = 1), and *Microviridae* (*n* = 108), others in putative families that, at present, remain unclassified (*n* = 20) and one DNA molecule that only encodes a replication associated protein. Among these novel viruses, two putative multi-component virus genomes were recovered, and these are most closely related to a Tongan flying fox faeces-associated multi-component virus. Given that the only other known multi-component circular replication-associated (Rep) protein encoding single-stranded (CRESS) DNA viruses infecting plants are in the families *Geminiviridae* (members of the genus *Begomovirus*) and *Nanoviridae*, it appears these are likely a new multi-component virus group which may be associated with animals. This study reiterates the diversity of ssDNA viruses in nature and in particular with the New Zealand blackflies.

## 1. Introduction

Hematophagous insects are responsible for vectoring a wide range of pathogens. Vectors of important mammalian and avian pathogens, such as mosquitoes, are members of the order Diptera (suborder: Nematocera). One group of blood sucking insects, commonly referred to as blackflies or sandflies, are members of the Simuliidae family [1]. Although blackflies are found globally, only some species consume a blood meal. For those species that feed on blood, it is the adult females that do so, whereas the males feed primarily on nectar. Aquatic environments are essential for the life cycle of blackflies, with the egg, larval and pupae stages all occurring in flowing water, followed by the emergence of a winged adult form. 

Blackflies are known to transmit a handful of parasites, predominantly protozoa and parasitic worms. In humans, the most important blackfly vectored pathogen is the parasitic nematode, *Onchocerca volvulus* which causes river blindness. Although rare, globally *O. volvulus* has a significant impact on human health in Africa, affecting over 18 million people [2,3]. Avian haemazoan parasites, such as those in the genus *Leucocytozoon*, are also commonly transmitted by blackflies, with different blackfly species showing preference to the bird species they bite and harbouring a variety of parasite linages [4,5]. The most studied arbovirus transmitted by blackflies is vesicular stomatitis virus (family *Rhabdoviridae*, genus *Vesiculovirus*) that typically infects livestock, however, zoonotic events have also been reported [6]. Invertebrate iridoviruses (family *Iridoviridae*), which are double-stranded DNA viruses, have been identified in blackflies across the globe, often causing a covert infection [7,8].

Several species of blackfly are endemic to New Zealand, two of which, the New Zealand blackfly (*Austrosimulium australense)* and the west coast blackfly (*A. ungulatum*), consume blood meals [9,10]. They are notorious for their persistence in pursuit of a blood meal and in various locations are present in overwhelming swarms. Despite having some knowledge on the ecology of New Zealand blackflies, we know very little about the viruses that are circulating in these insects. No human pathogens vectored by blackflies in New Zealand have been documented and, with the exception of protozoal transmission in some avian species, little is known about the microorganisms circulating in these insects.

In recent years, studies have used viral metagenomics as a non-biased approach for identifying viruses circulating in hematophagous and phytophagous insects [11,12,13,14,15]. This has resulted in the identification of a vast number of viruses, circulating in the hosts these insects are feeding on, from the surrounding environment, and those that infect the insects themselves. Arthropods have been broadly shown to harbour a wide range of viruses with circular replication-associated (Rep) protein encoding single-stranded (CRESS) DNA genomes [11,13,14,16,17]. At present there are many established CRESS DNA virus families; *Bacillidnaviridae*, *Circoviridae*, *Geminiviridae*, *Genomoviridae*, *Nanoviridae*, and *Smacoviridae*, all of whose members encode Reps that share conserved replication associated motifs, an origin of replication and are usually <6 kb in size [18]. Also, viruses in the family *Microviridae* which infect bacteria, typically encode a major capsid, minor capsid and a replication initiation protein, and range in size from 4–7 kb [19]. In addition, numerous novel CRESS DNA viruses have been identified in arthropods which are yet to be taxonomically classified [11,20]. 

Viral metagenomic studies reveal a great deal about viruses circulating in arthropods. For example, a study on mosquitoes [14] identified viral sequences with similarities to animal-infecting ssDNA viruses (families: *Anelloviridae*, *Circoviridae*, *Parvoviridae*), double-stranded (ds) DNA viruses (families: *Herpesviridae*, *Poxviridae* and *Papillomaviridae*), plant-infecting ssDNA viruses (families: *Geminiviridae* and *Nanoviridae*), and three bacteria-infecting dsDNA viruses (families: *Myoviridae*, *Podoviridae* and *Siphoviridae*). Viral metagenomic studies allows for a snapshot of the viruses circulating in the vectors hosts, the insect and surrounding ecosystem to be identified. Based on the lack of information on the widespread New Zealand hematophagous insect commonly known as the blackfly, we undertook a metagenomics approach to investigate the associated ssDNA virome.

## 2. Materials and Methods

### 2.1. Collection of Blackflies and Isolation of Viral Nucleic Acid

For this project, 40 individual blackflies were collected from North Canterbury, New Zealand in 2015. The 40 individuals were collected from a single site. The sex of the individuals, and whether they had consumed a blood meal, was determined. These samples were pooled and homogenized using a pestle in 2 mL of SM buffer (0.1 M NaCl, 50 mM Tris/HCl-pH 7.4, 10 mM MgSO_4_). The homogenized sample was centrifuged for 10 min at 10,000 rpm and the resulting supernatant was filtered through a 0.2 µM filter. The viral particles in the filtrate were precipitated overnight at 4 °C with 15% PEG and following this the solution was centrifuged at 14,000 rpm for 10 min and resulting pellet resuspended in 500 µL of SM buffer. Following this, 200 µL of the resuspended material was subsequently used to isolate viral DNA using the High Pure Viral Nucleic Acid Kit (Roche Diagnostics, USA) according to the manufacturer’s specifications. The viral nucleic acid was then used in a rolling circle amplification reaction with the TempliPhi™ kit (GE Healthcare, USA) to preferentially amplify circular DNA molecules. 

### 2.2. High-Throughput Sequencing and Viral Genome Verification

Rolling circle amplified DNA was used to prepare 2 × 150 bp paired-end libraries for sequencing on an Illumina 2500 platform at Macrogen Inc. (Korea). The paired-end reads were *de novo* assembled using metaSPAdes v 3.12.0 [21] and resulting contigs (>750 nts) were filtered for viral-like sequences using BLASTx [22] against viral protein database generated from the GenBank RefSeq depository. For contigs with similarities to viruses in the *Microviridae* family, full *de novo* assembled genomes were confirmed by mapping raw reads using BBMap [23], and deemed credible with a coverage level of greater than 10×. For viral contigs with similarities to other ssDNA viruses, abutting primers (Table S1) were designed to recover the complete genomes by PCR using Kapa HiFi HotStart DNA polymerase (KAPA Biosystems, USA). Amplicons were resolved on a 0.7% agarose gel, the correct size amplicons were excised, gel purified and cloned into pJET1.2 cloning vector (ThermoFisher Scientific, USA). Recombinant plasmids containing the viral genomes were purified from transformed XL blue *E. coli* competent cells and Sanger sequenced at Macrogen Inc. (Korea) by primer walking. The Sanger sequences were assembled using Geneious software V11.1.5.

### 2.3. Network Construction, Phylogenetic and Similarity Comparison Analyses

The blackfly viral *rep* (CRESS DNA viruses) and the *major capsid protein* (*mcp*) gene (microviruses) together with those available in GenBank were extracted, translated and used to build a Rep and MCP protein sequence dataset. The Rep of circoviruses, geminiviruses, smacoviruses, genomoviruses and nanoviruses and the MCP of viruses in the subfamily *Gokushovirinae* (family *Microvividae*) clustered first with CD-HIT [24] using 0.9 sequence identity cut off and a representative from each cluster was included in the final dataset. The Rep and MCP protein sequences were used separately to build sequence similarity networks (E-value = 1 × 10^−5^) using the EFI-Enzyme similarity tool server [25,26]. The MCP similarity network was constructed using minimum similarity score 10^−175^ and the Rep with 10^−60^. The score is the similarity threshold for connected nodes, i.e., proteins, with each other and thus those with scores below this value are not connected. Protein similarity networks were visualised using Cytoscape V3.7.0 [27].

Based on the network clusters, unique cluster sequences datasets were built for each major cluster (comprised of ten or more members) that contained a blackfly derived sequence. The datasets included genomoviruses, circoviruses and five additional clusters labelled cluster group 1–5 for the unclassified CRESS DNA viruses and two microvirus groups labelled cluster MV group 1 and 2. The sequences in each of these cluster groups were aligned using MUSCLE [28] and maximum likelihood phylogenetic trees inferred using PHYML [29] with the best fit models, determined using ProtTest [30]. The substitution models used are genomoviruses, LG+I+G; circoviruses, rtREV+G+I; CRESS group 1, rtREV+G+I; CRESS group 2, WAG+G+I; CRESS group 3, rtREV+G; CRESS group 4, WAG+G+I; CRESS Group 5, WAG+G+I, MV group 1, rtRev+G+I; MV group 2, rtREV+G+I+F. Branches with aLRT support of <0.8 were collapsed using TreeGraph2 [31]. The Maximum likelihood phylogenetic trees were midpoint rooted with the exception of the genomoviruses which were rooted using the geminivirus Reps and the cycloviruses with circoviruses Reps.

BLASTx [22] comparisons were undertaken for any singletons to determine the most closely related sequences in GenBank. For clusters comprised of less than four sequences an amino acid pairwise comparison using SDT V1.2 [32] was undertaken.

## 3. Results and Discussion

**Figure 1:**
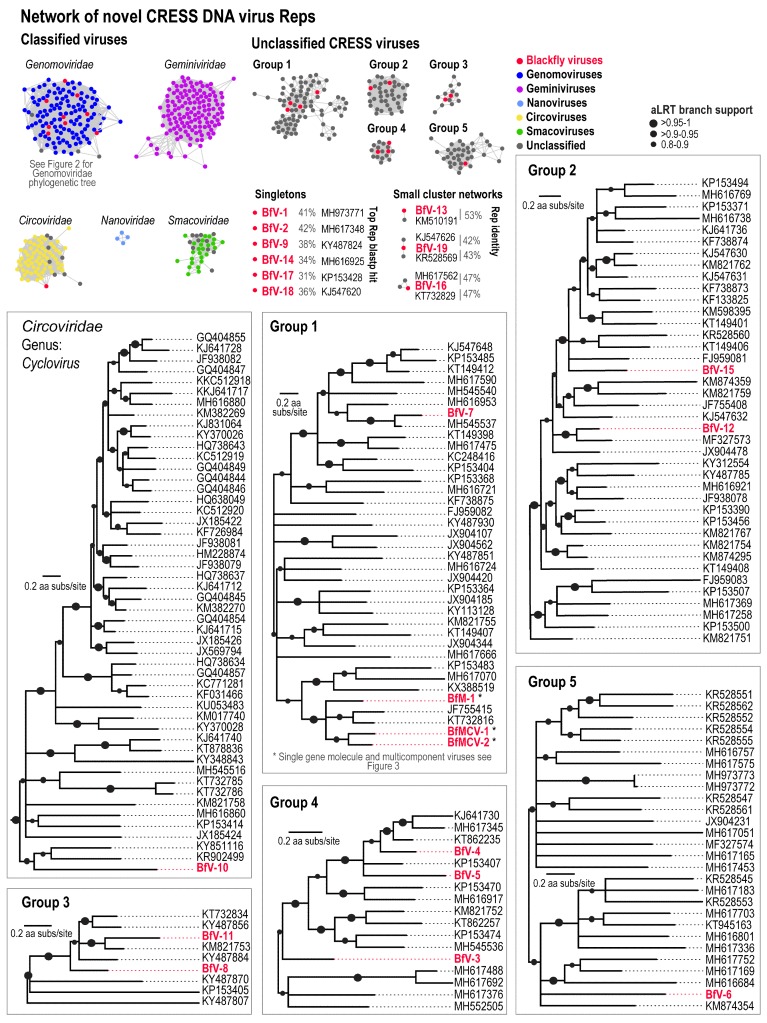
A network and phylogenetic analyses of the circular replication-associated (Rep) protein encoding single-stranded (CRESS) DNA virus replication-associated (Rep) protein sequences. The network analyses shows the clustering of the blackfly CRESS DNA viruses (shown as red circles) within taxonomic family groupings, larger and smaller groupings of unclassified CRESS DNA viruses (only those groups containing CRESS DNA viruses from this study), and singletons from this study that do not cluster with other known Reps. Closest Rep comparisons and percentage similarity are shown for the smaller groups and singletons. Maximum likelihood phylogenetic trees of the Rep sequence of circovirus (showing only the *Cyclovirus* genus clade) and the five larger unclassified CRESS DNA virus groupings. Blackfly originating sequence names are shown in red. The maximum likelihood phylogenetic tree of the Rep sequence for the genomoviruses is shown in Figure 2.

In a pool of 40 individual blackflies, diverse CRESS DNA viruses were identified. Cluster analyses using the Rep protein, which is the most conserved gene among CRESS DNA viruses, provides a broad overview of the extensive range of viruses recovered in the blackfly samples (Table 1 and Figure 1). The network analyses results reveal (Figure 1) that the Reps from the blackfly derived viruses or DNA molecules cluster with those in the *Genomoviridae* family (n = 9), *Circoviridae* family (n = 1), unclassified CRESS DNA viruses (n = 15) or as singletons (n = 6).

### 3.1. Blackfly CRESS DNA Viruses Clustering with Genomoviruses

**Figure 2:**
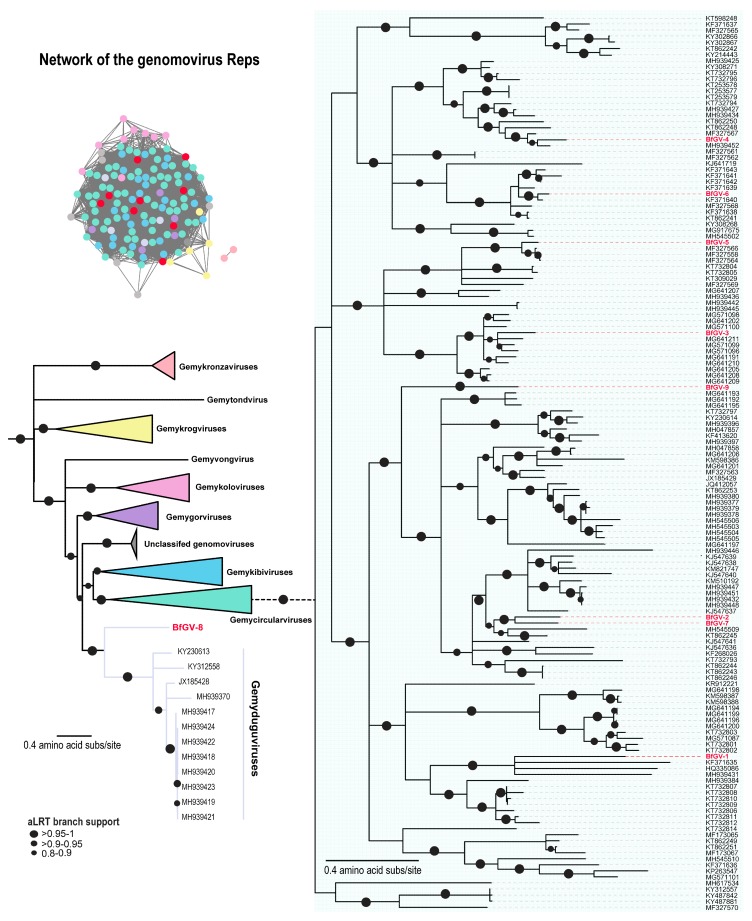
Network and phylogenetic analysis of the Rep sequences of genomovirus, each genus with a corresponding colour and blackfly originating genomoviruses shown in red.

Genomoviruses have been frequently found to be associated with various arthropods [11,14,16,17,20,33,34] in addition to various other samples. Genomoviruses are typically ~2 kb in genome size and likely infect fungi based on a study which showed a genomovirus species, *Sclerotinia sclerotiorum hypovirulence virus 1*, is able to replicate and cause hypovirulence in *Sclerotinia sclerotiorum* [35]. Taxonomically, genomoviruses group in nine genera; *Gemycircularvirus*, *Gemyduguivirus*, *Gemygorvirus*, *Gemykibivirus*, *Gemykolovirus*, *Gemykrogvirus*, *Gemykronzavirus*, *Gemytondvirus and Gemyvongvirus.* Here we describe nine novel genomoviruses hereby referred to as blackfly genomovirus (BfGV) 1–9 (Table 1, Figure 1 and Figure 2). Eight of these (BfGV 1–7, -9) can be more broadly classified into the *Gemycircularvirus* genus and BfGV-8 Rep lies basal to that of the members of *Gemyduguivirus* genus (Figure 2). Based on the 78% full genome species demarcation [36] the seven of the genomoviruses, BfGV-8 whose Rep is basal to those of gemyduguiviruses and six (BfGV-1, -2, -4, -5, -9) assigned to the *Gemycircularvirus* genus, are new species. The genome of BfGV-3 shares 79% identity with a genomovirus from snowshoe hare faeces (MG611211; [37]) and BfGV-6 shares 81% identity with a genomovirus from porcine faeces (KF371640; [38]. All share a conserved nonanucleotide “TAATATTAT” sequence which is nicked by Rep to initiate replication. The Reps of these genomoviruses share between 33–95% amino acid identities with those of all genomoviruses whereas the capsid proteins (CPs) share 29–79% identity. (Supplementary Data 1). Replication associated motifs in the Rep are conserved throughout the nine genomovirus genomes (Table S2).

### 3.2. Divergent CRESS DNA Viruses

A large cohort of Rep-encoding viruses exist that are divergent and do not, at present, fall into classified viral families. These vary in genome organisation but all encode a Rep and putative CP. Several novel CRESS DNA viruses were also discovered in this study, all but one cluster outside major virus family groups and are distributed across five larger network clusters and three smaller clusters contain two to three sequences (Table 1, Figure 1). Nine do not cluster with any other Reps therefore are represented as singletons (Table 1, Figure 1). These range in genome size from ~1.8 to 3 kb and are referred to as blackfly DNA virus (BfV) 1–19 (Table 1). Genome organisation is highly variable, see Table 1 for details on open-reading frame orientations. Interestingly, BfV-18 appears to have an unusual *rep* which contains two predicted intron regions. All Rep encoding molecules harbour RCR and SF3 helicase motifs, with the exception of BfV-1 and -2 which are apparently missing motif C and RCR motif II, respectively.

The Rep of BfV-7 in group 1 is most closely related to that from a giant house spider (MH545537) [11], sharing 77% Rep identity (Sup data 2). In group 2, the Reps of BfV-12 and -15 share 70% identity with each other, and 68% with giant panda circovirus 1 Rep (MF327573) and 60% with dragonfly larvae-associated circular virus-2 Rep (KF738874) [39], respectively. Group 3 includes Reps of BfV-8 which shares 68% with those of Pacific flying fox faeces-associated circular DNA virus-15 (KT732834) [40] and BfV-11 which shares 70% with sewage-associated circular DNA virus-18 (KM821753) [41]. Reps of BfV-3, -4 and -5 in group 4 share 62% identity with CRESS virus from a minnow (MH617376) [42], and 76% and 68% with llama faeces-associated circular DNA virus-1 (KT862235) [43], respectively. The Rep of BfV-6 in group 5 shares 48% identity with that of Avon-Heathcote estuary-associated circular virus 24 (KM874354) [44].

Several viruses related to those in the family *Circoviridae* have also been identified in arthropods [11,14,15,45,46,47,48]. Here we identify a blackfly DNA virus (BfV-10) whose Rep clusters with members of the *Circoviridae* family and it is phylogenetically basal to the cycloviruses (Figure 1). The Rep of BfV-10 share 35–43% amino acid identity to Reps of circoviruses.

Reps of BfV-13, -19 and -16 form small clusters sharing 42–53% identities to their closest related Reps from Bromus-associated circular DNA virus 2 (KM510191) [49], *Lytechinus variegatus* variable sea urchin-associated circular virus (KR528569) [50], sewage-associated circular DNA virus-2 (KJ547626) [41], CRESS virus sp. isolate ctbg173 (MH617562) [42], Pacific flying fox faeces-associated circular DNA virus-2 (KT732829) [40]. BfV-1, -2, -9, -14, -17 and -18 Reps are singletons sharing 31–42% with Reps of *Apis mellifera* virus 15 (MH973771) [34], Circoviridae sp. isolate ctbe41 (MH617348) [42], uncultured virus clone CG155 (KY487824) [51], CRESS virus sp. isolate ctdb65 (MH616925) [42], Lake Sarah-associated circular virus-18 (KP153428) [44] and sewage-associated circular DNA virus-1 (KJ547620) [41] (Figure 1).

### 3.3. Multi-Component Viruses and Circular Rep-Encoding DNA Molecule

**Figure 3:**
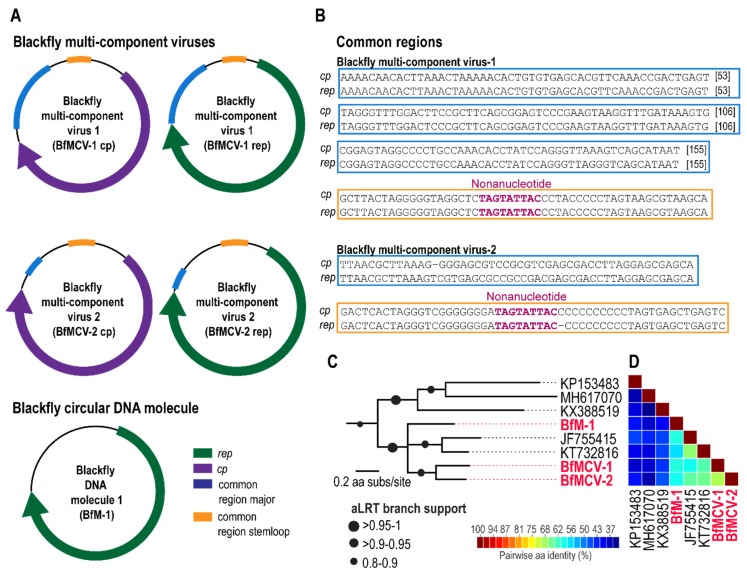
(**A**) Genome organisation of two blackfly multicomponent DNA viruses (1 and 2) and a DNA molecule indicating open reading frames and common regions (**B**) Alignment of the common regions from blackfly multicomponent virus 1 and 2 (**C**) Phylogeny of Rep for the two multicomponent viruses, DNA molecule and closest relatives (**D**) Pairwise comparison of the Reps show percentage similarity.

Viruses in the *Nanoviridae* family infect plants and are comprised of up to eight separate circular DNA components, all encoding a different functional gene including a Rep. Members of another plant infecting virus family, *Geminiviridae*, are known to have satellite molecules which enhance replication and pathogenicity. More recently a multi-component virus was isolated from faecal samples of Pacific flying fox, this, like other multi-component DNA viruses shares sequence recognition regions in the intergenic regions. In this study, three circular DNA molecules that encode a *rep* gene were identified. For two of these, cognate molecules encoding a *cp* gene were identified and therefore these represent two multicomponent viruses (Table 1, Figure 3A–C, Supplementary Data 2). These components have common regions in the intergenic region (Figure 3B), such common regions in multicomponent viruses act as recognition sites for initiation of replication [52]. These, referred to as blackfly multicomponent virus (BfMCV) 1 and 2, have Reps most similar to another multicomponent virus recovered from a Pacific flying fox multicomponent virus (KT732816) [40], sharing 65% and 67% aa similarity, respectively (Figure 3C,D). The cognate BfMCV 1 and 2 CPs share 44% with leaf-footed bug circular genetic element (MH545544) [11] and 43% with Pacific flying fox multicomponent virus (KT732816) [40]. No cognate molecule was identified for the third DNA molecule, referred to as Blackfly DNA molecule 1, however, this molecule is most closely related to the other two multicomponent viruses detected here, sharing 61–62% Rep identity, and to rodent stool associated circular genome virus (JF755415) [53] sharing 62% Rep identity. A cognate molecule encoding a CP may therefore not have been recovered, although no molecule was identified in the high-throughput sequencing data, or this genetic element may represent replication. All molecules have the same nonanucleotide sequence ‘TAGTATTAC’.

### 3.4. Bacteria-Infecting CRESS DNA Viruses

**Figure 4:**
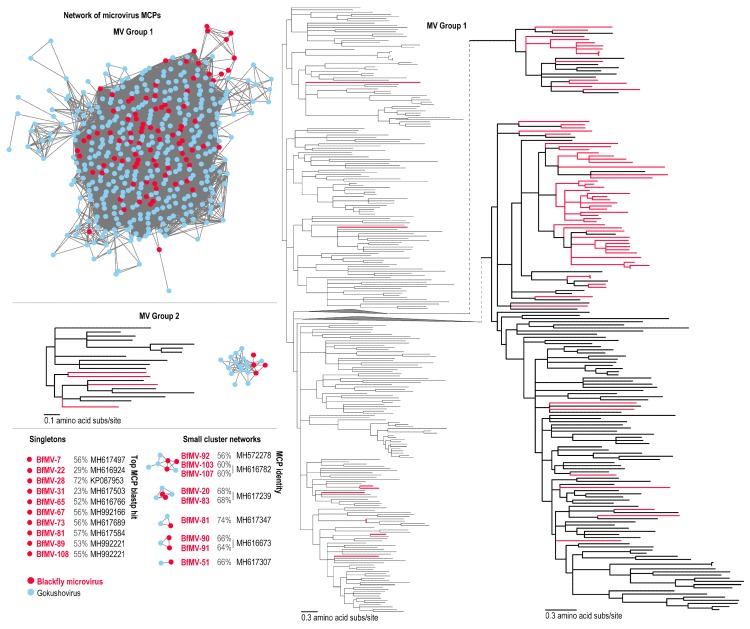
Network analyses of the microvirus major capsid protein. Maximum likelihood phylogeny of the MCP sequences of microviruses for the two major groups (MV group 1 and 2) are shown. Smaller networks and singletons are shown with closet Rep comparisons and percentage similarities. Blackfly derived sequence are shown in red.

Microviruses, which infect bacteria, are commonly found where their host is present including the microbiome of arthropods [34,54] and harbour unidirectional genomes which encode a replication initiation protein, major and minor capsid proteins and other accessory proteins [19]. Divided into two subfamilies, *Gokushovirinae* and *Bullavirinae* (International committee for virus taxonomy: 2018 release; https://talk.ictvonline.org/taxonomy/), microviruses are largely host specific [19]. Here, we identify 108 novel microviruses from blackflies. Using the most well conserved protein, the MCP, we show the wide scope of microvirus diversity present in blackflies (Figure 4). A large proportion of blackfly microviruses (BfMVs) MCPs group with those of gokushoviruses (Figure 4). The MCPs of 88 BfMVs cluster in MV group 1, four in MV group 2 and the remaining are part of small network clusters or are singletons. Although a few BfMVs in the MV group 1 are interspersed throughout the MCP phylogeny, the majority fall in two major clades (Figure 4), sharing between 51–85% MCP identity with other microviruses (Supplementary Data 3). The MCPs of BfMVs in MV group 2, along with the other small network clusters, share 29–74% identity with those of other microviruses (Figure 4 and Supplementary Data 3). The singletons range in similarity to nearest neighbours in the public database sharing between 23–72% MCP protein sequence identity (Figure 4 and Supplementary Data 3).

Many of the newly described microviruses identified in this study group with those isolated from other arthropods such as those from honey bees [34]. Taking into consideration that only 40 blackflies were sampled, the sheer number and diversity of microviruses that have been found here is remarkable. This may, in fact, reflect an equally diverse associated bacterial community in the blackfly and the animals from which they obtain a blood meal. Furthermore, this, taken with the recent recovery of many novel microviruses from arthropods [34,54], indicates the presence of distinct arthropod microvirus populations.

## 4. Conclusions

The ongoing expansion of our knowledge of the CRESS DNA viruses facilitated by high-throughput sequencing approaches emphasizes the breadth of diversity in various ecosystems and organisms. The diversity and number of CRESS DNA viruses associated with arthropods alone is staggering [11,14,16,20,48]. Here, we report the identification of CRESS DNA viruses associated with blackflies in New Zealand. Nine genomoviruses, 19 unclassified CRESS DNA viruses, two multicomponent viruses, a circular rep-encoding DNA molecule and 108 microviruses. To date, the only multicomponent CRESS viruses in established families are the nanoviruses and geminiviruses which both infect plants. Other than these, our group have identified novel multicomponent viruses previously in faecal matter of Pacific flying foxes [40] and now in blackflies. These appear to be related (i.e., CP and Reps) and thus eludes to the fact that there are other Rep encoding multicomponent viruses [40], given the presence of a completely unique group comprised of the BfMCVs and the pacific flying fox multicomponent virus beyond those that belong to the *Geminiviridae* and *Nanoviridae* families. Due to the blood feeding nature of blackflies, these viruses may have originated from the blackflies, the hosts they feed from or surrounding environment. Although further investigation is needed to truly determine the hosts of the CRESS viruses, we know the microviruses infect bacteria and therefore, prominent groups of related microviruses may reflect a specific blackfly bacterial profile. This study provides knowledge on viruses associated with the understudied hematophagous blackfly of New Zealand and further displays the sheer breadth of CRESS DNA viral diversity in arthropods globally.

## Figures and Tables

**Table 1:** Summary of the genomes of blackfly associated viruses including genome organisation and GenBank Accession numbers.

Family/Subfamily	Genus	Virus Name	Accession Number	Size (nts)	ORF Orientation
*Genomoviridae*	*Gemycircularirus*	Blackfly genomovirus-1 SF02 506	MK433242	2290	Bidirectional
		Blackfly genomovirus-2 SF02 631	MK433234	2131	Bidirectional
		Blackfly genomovirus-3 SF02 1766	MK433235	2138	Bidirectional
		Blackfly genomovirus-4 SF02 836	MK433236	2181	Bidirectional
		Blackfly genomovirus-5 SF02 599	MK433237	2182	Bidirectional
		Blackfly genomovirus-6 SF02 459	MK433238	2186	Bidirectional
		Blackfly genomovirus-7 SF02 767	MK433239	2195	Bidirectional
		Blackfly genomovirus-9 SF02 507	MK433241	2217	Bidirectional
*Genomoviridae*	*Gemyduguvirus*	Blackfly genomovirus-8 SF02 579	MK433240	2226	Bidirectional
Unclassified CRESS DNA virus	Unassigned	Blackfly DNA virus-1 SF02 666	MK433215	1805	Unidirectional
		Blackfly DNA virus-2 SF02 583	MK433216	1936	Bidirectional
		Blackfly DNA virus-3 SF02 402	MK433217	2005	Unidirectional
		Blackfly DNA virus-4 SF02 664	MK433218	2047	Unidirectional
		Blackfly DNA virus-5 SF02 839	MK433219	2058	Unidirectional
		Blackfly DNA virus-6 SF01 308	MK433220	2172	Bidirectional
		Blackfly DNA virus-7 SF02 462	MK433221	2306	Bidirectional
		Blackfly DNA virus-8 SF02 1137	MK433222	2337	Bidirectional
		Blackfly DNA virus-9 SF02 881	MK433223	2389	Bidirectional
		Blackfly DNA virus-10 SF02 899	MK433224	2444	Bidirectional
		Blackfly DNA virus-11 SF02 963	MK433225	2449	Bidirectional
		Blackfly DNA virus-12 SF02 422	MK433226	2466	Unidirectional
		Blackfly DNA virus-13 SF02 413	MK433227	2501	Bidirectional
		Blackfly DNA virus-14 SF02 295	MK433228	2583	Bidirectional
		Blackfly DNA virus-15 SF02 403	MK433229	2697	Bidirectional
		Blackfly DNA virus-16 SF02 377	MK433230	2704	Bidirectional
		Blackfly DNA virus-17 SF02 1426	MK433231	2835	Unidirectional
		Blackfly DNA virus-18 SF02 66	MK433232	3003	Bidirectional
		Blackfly DNA virus-19 SF02 380	MK433233	2002	Bidirectional
Unclassified Circular DNA molecules	Unassigned	Blackfly DNA molecule 1 - rep	MK561604	1157	Unidirectional
Unclassified Multi-component virus	Unassigned	Blackfly multicomponent virus 1 - rep	MK561605	1163	Unidirectional
		Blackfly multicomponent virus 1 - cp	MK561606	1154	Unidirectional
		Blackfly multicomponent virus 2 - rep	MK561607	1136	Unidirectional
		Blackfly multicomponent virus 2 - cp	MK561608	1133	Unidirectional
*Microviridae*/*Gokushovirinae*	Unassigned	Blackfly microvirus-1_ SF02_SP_9	MK249160	4873	Unidirectional
		Blackfly microvirus-2_ SF02_SP_11	MK249161	4544	Unidirectional
		Blackfly microvirus-3_ SF02_SP_12	MK249162	4625	Unidirectional
		Blackfly microvirus-4_ SF02_SP_13	MK249163	4660	Unidirectional
		Blackfly microvirus-5_ SF02_SP_14	MK249164	4594	Unidirectional
		Blackfly microvirus-6_ SF02_SP_15	MK249165	4675	Unidirectional
		Blackfly microvirus-7_ SF02_SP_17	MK249166	4379	Unidirectional
		Blackfly microvirus-8_ SF02_SP_18	MK249167	4483	Unidirectional
		Blackfly microvirus-9_ SF02_SP_20	MK249168	4324	Unidirectional
		Blackfly microvirus-10_ SF02_SP_21	MK249169	4656	Unidirectional
		Blackfly microvirus-11_ SF02_SP_22	MK249170	4565	Unidirectional
		Blackfly microvirus-12_ SF02_SP_24	MK249171	4231	Unidirectional
		Blackfly microvirus-13_ SF02_SP_25	MK249172	4582	Unidirectional
		Blackfly microvirus-14_ SF02_SP_27	MK249173	4694	Unidirectional
		Blackfly microvirus-15_ SF02_SP_28	MK249174	4557	Unidirectional
		Blackfly microvirus-16_ SF02_SP_30	MK249175	4689	Unidirectional
		Blackfly microvirus-17_ SF02_SP_31	MK249176	4474	Unidirectional
		Blackfly microvirus-18_ SF02_SP_32	MK249177	4841	Unidirectional
		Blackfly microvirus-19_ SF02_SP_34	MK249178	4727	Unidirectional
		Blackfly microvirus-20_ SF02_SP_36	MK249179	4498	Unidirectional
		Blackfly microvirus-21_ SF02_SP_38	MK249180	4461	Unidirectional
		Blackfly microvirus-22_ SF02_SP_44	MK249181	6565	Unidirectional
		Blackfly microvirus-23_ SF02_SP_45	MK249182	4565	Unidirectional
		Blackfly microvirus-24_ SF02_SP_46	MK249183	4633	Unidirectional
		Blackfly microvirus-25_ SF02_SP_49	MK249184	4521	Unidirectional
		Blackfly microvirus-26_ SF02_SP_50	MK249185	4731	Unidirectional
		Blackfly microvirus-27_ SF02_SP_51	MK249186	4581	Unidirectional
		Blackfly microvirus-28_ SF02_SP_52	MK249187	4667	Unidirectional
		Blackfly microvirus-29_ SF02_SP_54	MK249188	4637	Unidirectional
		Blackfly microvirus-30_ SF02_SP_57	MK249189	4980	Unidirectional
		Blackfly microvirus-31_ SF02_SP_67	MK249190	4901	Unidirectional
		Blackfly microvirus-32_ SF02_SP_69	MK249191	4633	Unidirectional
		Blackfly microvirus-33_ SF02_SP_71	MK249192	4901	Unidirectional
		Blackfly microvirus-34_ SF02_SP_73	MK249193	4870	Unidirectional
		Blackfly microvirus-35_ SF02_SP_74	MK249194	4765	Unidirectional
		Blackfly microvirus-36_ SF02_SP_75	MK249195	4789	Unidirectional
		Blackfly microvirus-37_ SF02_SP_78	MK249196	4774	Unidirectional
		Blackfly microvirus-38_ SF02_SP_79	MK249197	4711	Unidirectional
		Blackfly microvirus-39_ SF02_SP_80	MK249198	4858	Unidirectional
		Blackfly microvirus-40_ SF02_SP_82	MK249199	4799	Unidirectional
		Blackfly microvirus-41_ SF02_SP_83	MK249200	4825	Unidirectional
		Blackfly microvirus-42_ SF02_SP_84	MK249201	4779	Unidirectional
		Blackfly microvirus-43_ SF02_SP_87	MK249202	4768	Unidirectional
		Blackfly microvirus-44_ SF02_SP_88	MK249203	4615	Unidirectional
		Blackfly microvirus-45_ SF02_SP_89	MK249204	4630	Unidirectional
		Blackfly microvirus-46_ SF02_SP_91	MK249205	4733	Unidirectional
		Blackfly microvirus-47_ SF02_SP_93	MK249206	4721	Unidirectional
		Blackfly microvirus-48_ SF02_SP_97	MK249207	4646	Unidirectional
		Blackfly microvirus-49_ SF02_SP_98	MK249208	4700	Unidirectional
		Blackfly microvirus-50_ SF02_SP_99	MK249209	4706	Unidirectional
		Blackfly microvirus-51_ SF02_SP_100	MK249210	4721	Unidirectional
		Blackfly microvirus-52_ SF02_SP_104	MK249211	4692	Unidirectional
		Blackfly microvirus-53_ SF02_SP_106	MK249212	4680	Unidirectional
		Blackfly microvirus-54_ SF02_SP_107	MK249213	4706	Unidirectional
		Blackfly microvirus-55_ SF02_SP_108	MK249214	4647	Unidirectional
		Blackfly microvirus-56_ SF02_SP_110	MK249215	4618	Unidirectional
		Blackfly microvirus-57_ SF02_SP_115	MK249216	4608	Unidirectional
		Blackfly microvirus-58_ SF02_SP_116	MK249217	4596	Unidirectional
		Blackfly microvirus-59_ SF02_SP_117	MK249218	4590	Unidirectional
		Blackfly microvirus-60_ SF02_SP_118	MK249219	4621	Unidirectional
		Blackfly microvirus-61_ SF02_SP_120	MK249220	4617	Unidirectional
		Blackfly microvirus-62_ SF02_SP_122	MK249221	4591	Unidirectional
		Blackfly microvirus-63_ SF02_SP_123	MK249222	4499	Unidirectional
		Blackfly microvirus-64_ SF02_SP_124	MK249223	4510	Unidirectional
		Blackfly microvirus-65_ SF02_SP_125	MK249224	4507	Unidirectional
		Blackfly microvirus-66_ SF02_SP_126	MK249225	4581	Unidirectional
		Blackfly microvirus-67_ SF02_SP_128	MK249226	4503	Unidirectional
		Blackfly microvirus-68_ SF02_SP_129	MK249227	4570	Unidirectional
		Blackfly microvirus-69_ SF02_SP_130	MK249228	4498	Unidirectional
		Blackfly microvirus-70_ SF02_SP_131	MK249229	4558	Unidirectional
		Blackfly microvirus-71_ SF02_SP_135	MK249230	4488	Unidirectional
		Blackfly microvirus-72_ SF02_SP_137	MK249231	4551	Unidirectional
		Blackfly microvirus-73_ SF02_SP_138	MK249232	4335	Unidirectional
		Blackfly microvirus-74_ SF02_SP_139	MK249233	4547	Unidirectional
		Blackfly microvirus-75_ SF02_SP_142	MK249234	4538	Unidirectional
		Blackfly microvirus-76_ SF02_SP_143	MK249127	4471	Unidirectional
		Blackfly microvirus-77_ SF02_SP_145	MK249128	4529	Unidirectional
		Blackfly microvirus-78_ SF02_SP_146	MK249129	4521	Unidirectional
		Blackfly microvirus-79_ SF02_SP_148	MK249130	4549	Unidirectional
		Blackfly microvirus-80_ SF02_SP_149	MK249131	4460	Unidirectional
		Blackfly microvirus-81_ SF02_SP_150	MK249132	4460	Unidirectional
		Blackfly microvirus-82_ SF02_SP_156	MK249133	4443	Unidirectional
		Blackfly microvirus-83_ SF02_SP_159	MK249134	4438	Unidirectional
		Blackfly microvirus-84_ SF02_SP_160	MK249135	4483	Unidirectional
		Blackfly microvirus-85_ SF02_SP_162	MK249136	4350	Unidirectional
		Blackfly microvirus-86_ SF02_SP_163	MK249137	4484	Unidirectional
		Blackfly microvirus-87_ SF02_SP_164	MK249138	4502	Unidirectional
		Blackfly microvirus-88_ SF02_SP_165	MK249139	4487	Unidirectional
		Blackfly microvirus-89_ SF02_SP_166	MK249140	4410	Unidirectional
		Blackfly microvirus-90_ SF02_SP_167	MK249141	4459	Unidirectional
		Blackfly microvirus-91_ SF02_SP_168	MK249142	4401	Unidirectional
		Blackfly microvirus-92_ SF02_SP_169	MK249143	4387	Unidirectional
		Blackfly microvirus-93_ SF02_SP_174	MK249144	4445	Unidirectional
		Blackfly microvirus-94_ SF02_SP_175	MK249145	4400	Unidirectional
		Blackfly microvirus-95_ SF02_SP_178	MK249146	4422	Unidirectional
		Blackfly microvirus-96_ SF02_SP_181	MK249147	4391	Unidirectional
		Blackfly microvirus-97_ SF02_SP_183	MK249148	4368	Unidirectional
		Blackfly microvirus-98_ SF02_SP_184	MK249149	4293	Unidirectional
		Blackfly microvirus-99_ SF02_SP_185	MK249150	4351	Unidirectional
		Blackfly microvirus-100_ SF02_SP_188	MK249151	4276	Unidirectional
		Blackfly microvirus-101_ SF02_SP_189	MK249152	4318	Unidirectional
		Blackfly microvirus-102_ SF02_SP_190	MK249153	4205	Unidirectional
		Blackfly microvirus-103_ SF02_SP_192	MK249154	4320	Unidirectional
		Blackfly microvirus-104_ SF02_SP_195	MK249155	4302	Unidirectional
		Blackfly microvirus-105_ SF02_SP_206	MK249156	4238	Unidirectional
		Blackfly microvirus-106_ SF02_SP_208	MK249157	4237	Unidirectional
		Blackfly microvirus-107_ SF02_SP_211	MK249158	4170	Unidirectional
		Blackfly microvirus-108_ SF02_SP_213	MK249159	4232	Unidirectional

## Supplementary Materials

The following are available online at https://www.mdpi.com/1999-4915/11/6/532/s1, Table S1: Primer details for each recovered blackfly virus genomes and molecules; Table S2: Rolling circle replication and helicase motifs identified in the Reps of the eukaryotic CRESS DNA viruses; Data S1: Pairwise identity comparison of the full genome, Rep and CP sequences of the genomoviruses recovered in this study together with all previously identified.
