# Supplementary material for: Unravelling the Single-Stranded DNA Virome of the New Zealand Blackfly
Source: Viruses. 2019 Jun 8;11(6):532. doi: 10.3390/v11060532 (PMC6630596; doi:10.3390/v11060532)
Supplement: Supplementary file 1 [file viruses-11-00532-s001.zip › Table S1.docx]

Table S1: Primer details for each recovered blackfly virus genomes and molecules

| **Viral grouping** | **Species grouping/Isolate name** | **Accession #** |  | **Forward primer** | **Reverse primer** |
| --- | --- | --- | --- | --- | --- |
| Genomovirus | Blackfly genomovirus-1 SF02 506 | MK433242 |  | CTTCACGACTTGGCAAGACACTACTTGCC | GTCCATATAGGACTAGACTCTTCGGCCTATC |
|  | Blackfly genomovirus-2 SF02 631 | MK433234 |  | CGATACCAGACTGCGATAGCCAGTTATCTC | GAAGTAGAGAGTCACCATTAGGTAAGTGACGC |
|  | Blackfly genomovirus-3 SF02 1766 | MK433235 |  | CTTGGCGAGATGTTTGGATGGAAATCTCCG | CAGAGGAACTCCTGAGGCAGGATACG |
|  | Blackfly genomovirus-4 SF02 836 | MK433236 |  | GTAAGAAGCGCGACACCATGGCCTAC | GACTTGTCATGTCGAGGATGGACCTCTTG |
|  | Blackfly genomovirus-5 SF02 599 | MK433237 |  | GACAATCACGCTGGCATCGGGCAAC | TTGTCATACTTGACTGTGACTCGTGAACTGTCTG |
|  | Blackfly genomovirus-6 SF02 459 | MK433238 |  | CAATGATACACTCAGCTCCAAGGAATGACAGG | GCAGAGAGTTGCACGCTGATGACGG |
|  | Blackfly genomovirus-7 SF02 767 | MK433239 |  | GAATAAGTGGACCCAGAGCTGACTGCG | GAAGTGGTCTGAGATTGAGAGTGCAGAGAG |
|  | Blackfly genomovirus-8 SF02 579 | MK433241 |  | GAAACGGTACCTCTCGAACGATATGATCGAC | ACGGAAGAGGATCTTGAACGTCTCTTCGGT |
|  | Blackfly genomovirus-9 SF02 507 | MK433240 |  | CGTCAACGGAGGCTCTGTCACCATTG | GTGCTAGTGTTGGCGTACTGCATCATGG |
| Unclassified CRESS DNA virus | Blackfly DNA Virus-1 SF02 666 | MK433215 |  | GTTCCCTTGTCTAGGTCTATGTCCAAATTCACAG | CGCACTGATCTAGAGATAATCCGTAACGATATGC |
|  | Blackfly DNA Virus-2 SF02 583 | MK433216 |  | GCCGGTAATACTAACGGCAGGGCTG | AGGGCGTTGGACTCAGACCCATCAG |
|  | Blackfly DNA Virus-3 SF02 402 | MK433217 |  | GGGATTGGATTCAGAGAATTGAACCAATCCAGC | GGCTCTTGGTACTTCAGTGGACATAACTTGC |
|  | Blackfly DNA Virus-4 SF02 664 | MK433218 |  | GGTTCCTCCGGACTTGCCTTCATTGATTG | CGTGATGAACAAGCCAGACAGTTCATGGC |
|  | Blackfly DNA Virus-5 SF02 839 | MK433219 |  | GAAGATCTTCCAGATGCATACATCAAGGAGCC | TTGATGTATGCATCTGGAAGATCTTCATGGGCC |
|  | Blackfly DNA Virus-6 SF01 308 | MK433220 |  | CAAGGATACCATATTGCAAGTACTGCATTTGGTCAC | AGTCTATCAAGGACCGACTCGTATTCTCCAC |
|  | Blackfly DNA Virus-7 SF02 462 | MK433221 |  | CAGGATGATACATCCATGATCCCAGGACAAC | ACCCTCACTCCAGTCGTTGATAGGATCTG |
|  | Blackfly DNA Virus-8 SF02 1137 | MK433222 |  | GATGGTATGGAGAGTACTCACTCCGAGG | CTCTGTTAGCAAGTGGTCCATAGCTTCTCG |
|  | Blackfly DNA Virus-9 SF02 881 | MK433223 |  | GTAGGCCTTAGAGACGAGTTTACCGGC | AGGGCCTACAAGGGTTCTAAGGGTTCAAC |
|  | Blackfly DNA Virus-10 SF02 899 | MK433224 |  | AGCTGTACGCGCATCTCGCTCTCG | GGCATGACACAGAAGAAGTGCCGGTTAG |
|  | Blackfly DNA Virus-11 SF02 963 | MK433225 |  | GTACAACAACATATTCAGGAGGGTATGGACCAC | GAGCTCTAAGGACTGCACTCGCTCTATTAAC |
|  | Blackfly DNA Virus-12 SF02 422 | MK433226 |  | CAAGCAGGATTGGTCAACAGAGTTGATGATGAC | TGCAGATCCTCGTTGGTAGATTGCGAAAGC |
|  | Blackfly DNA Virus-13 SF02 413 | MK433227 |  | GCAGAACACATCCACCGGAATCGTCAAATC | CACTGGCTGAGTACAGAGGTGGCTC |
|  | Blackfly DNA Virus-14 SF02 295 | MK433228 |  | GACTTACGGCAGAAGAGTGGCTCAATCTTC | TTACCTCGGGATTTAGACGTTCTACGATTCGATC |
|  | Blackfly DNA Virus-15 SF02 403 | MK433229 |  | GAACAACTTGAGCATGAACATCATGAGCAAACTCAAG | ATGCTGATACTGCTGGTGCAACTCCATTACC |
|  | Blackfly DNA Virus-16 SF02 377 | MK433230 |  | GATCGACACACGTGCATACGTTCACCAAC | CAGCTGGGATCGCAAGTGTCGTAGTC |
|  | Blackfly DNA Virus-17 SF02 1426 | MK433231 |  | GTACACACGGTGCCAATTTGCAAATGTGACG | ATTTGCTCTTCCAAGCATCCATTCGACTGGTATC |
|  | Blackfly DNA Virus-18 SF02 66 | MK433232 |  | GTAGTCGATGCATCGCTGCCAGTCG | GTCGCCAAAGATGGACATTTTCGAGAGCAC |
|  | Blackfly DNA Virus-19 SF02 380 | MK433233 |  | CAAGAACCCAAGGTACTTCGACTTCCGAG | ATCTCCAACTTTCTGGTTCCGGTGAAGAGG |
| Circular DNA molecule | Blackfly DNA molecule 1 - rep | MK561604 |  | GTGTGCGGTGTGCTGCCGACTATTC | TAGGGGTATGGGTAATACTAGTAGCCATACCG |
| Multi component virus | Blackfly multicomponent virus 1 - rep | MK561605 |  | GCTCACTATGAACCCTCACGCGGTAACCCTG | ACGAGGGAGCATCTCCTTCATAGTAGTAAATCC |
|  | Blackfly multicomponent virus 1 - cp | MK561606 |  | GATATCGATTTGAGGTCTCTCTGCAGGATACTC | CACAGGATACGATTGAGTATTGACTACCAGCATAG |
|  | Blackfly multicomponent virus 2 - rep | MK561607 |  | GGATTACTCTCTCCCCTATGGGTGATCAG | GATCTTCAGCGAGTACGACGAACCTCTC |
|  | Blackfly multicomponent virus 2 - cp | MK561608 |  | GTATTATGGCGATACGGGTACTGGGAAGTC | CACTCATTGATAATATCGTCATTATCAGGAGGCATTGG |
