# Supplementary material for: Unravelling the Single-Stranded DNA Virome of the New Zealand Blackfly
Source: Viruses. 2019 Jun 8;11(6):532. doi: 10.3390/v11060532 (PMC6630596; doi:10.3390/v11060532)
Supplement: Supplementary file 1 [file viruses-11-00532-s001.zip › Table S2.docx]

Table S2: Rolling circle replication and helicase motifs identified in the Reps of the eukaryotic CRESS DNA viruses;

| Viral grouping | Species grouping/Isolate name | Accession # | RCR Motifs | | | SF3 Helicase Motifs | | |
| --- | --- | --- | --- | --- | --- | --- | --- | --- |
|  |  |  | **I** | **II** | **III** | **Walker-A** | **Walker-B** | **Motif C** |
| Genomovirus | Blackfly genomovirus-1 SF02 506 | MK433242 | LLTYPQ | VHFHV | YVCK | GPSRLGKT | VFDDI | VSNT |
|  | Blackfly genomovirus-2 SF02 631 | MK433234 | LLTYSQ | THLHV | YAIK | GESRTGKT | VFDDI | ISNT |
|  | Blackfly genomovirus-3 SF02 1766 | MK433235 | LVTYSH | VHYHV | YAIK | GPTRLGKT | VFDDI | IANT |
|  | Blackfly genomovirus-4 SF02 836 | MK433236 | LLTYAQ | THLHV | YAVK | GASRLGKT | VFDDM | LANS |
|  | Blackfly genomovirus-5 SF02 599 | MK433237 | LLTYAH | FHFHV | YATK | GPTRMGKT | VIDDI | CSNE |
|  | Blackfly genomovirus-6 SF02 459 | MK433238 | LLTYAQ | THLHA | YATK | GDTRLGKT | VFDDM | LSNR |
|  | Blackfly genomovirus-7 SF02 767 | MK433239 | LLTYSQ | THLHV | YAIK | GESRTGKT | VFDDL | LSNV |
|  | Blackfly genomovirus-8 SF02 579 | MK433240 | LLTYSQ | IHFHA | YATK | GPSRTGKT | VFDDI | LCNN |
|  | Blackfly genomovirus-9 SF02 507 | MK433241 | LITYAQ | LHHHV | YAIK | GASRLGKT | VFDDI | LANV |
| Unclassified CRESS DNA virus | Blackfly DNA Virus-1 SF02 666 | MK433215 | CFTVHS | KHLQC | YCQE | DESTIEKI | QWEDF | ---- |
|  | Blackfly DNA Virus-2 SF02 583 | MK433216 | LKILTV | ----- | YVGE | DPLPFSSS | VEDSS | LPIA |
|  | Blackfly DNA Virus-3 SF02 402 | MK433217 | CFTLNN | PHLQG | YCSK | GEPGVGKS | IIDDF | VTSN |
|  | Blackfly DNA Virus-4 SF02 664 | MK433218 | CFTLNN | PHLQG | YCSK | GKPGVGKS | IIDDF | VTSN |
|  | Blackfly DNA Virus-5 SF02 839 | MK433219 | VFTFNN | PHLQG | YCIK | GPPGVGKS | IIDDF | VTSN |
|  | Blackfly DNA Virus-6 SF01 308 | MK433220 | VFTWNN | PHLQG | YSIK | NLAATLAL | KRDDL | MSVT |
|  | Blackfly DNA Virus-7 SF02 462 | MK433221 | CFTKNN | PHLQG | YCSK | GESGAGKT | LLDDV | VTSQ |
|  | Blackfly DNA Virus-8 SF02 1137 | MK433222 | ILTFPQ | PHLHV | YVTK | GKPNLGKT | VLDEF | LSNY |
|  | Blackfly DNA Virus-9 SF02 881 | MK433223 | FLTYSQ | RHLHA | YAKK | GKPGIGKT | IFDDL | LGNE |
|  | Blackfly DNA Virus-10 SF02 899 | MK433224 | CFTINN | KHIQG | YCSK | GLPGVGKS | VLDDF | FSSN |
|  | Blackfly DNA Virus-11 SF02 963 | MK433225 | FLTYAQ | PHLHV | YTIK | GPKNLGKT | VFDEF | ILSN |
|  | Blackfly DNA Virus-12 SF02 422 | MK433226 | ILTISQ | RHWQV | YVWK | GSTGTGKS | VFDEF | ITSN |
|  | Blackfly DNA Virus-13 SF02 413 | MK433227 | CFTCFG | EHWQG | YCKK | GATRLGKT | IFDDI | LTNF |
|  | Blackfly DNA Virus-14 SF02 295 | MK433228 | CLTIHV | YHWQM | YCSK | DPNGAGKS | VLLDL | FSNS |
|  | Blackfly DNA Virus-15 SF02 403 | MK433229 | MLTIPH | LHWQI | YVWK | GPTGTGKS | IIDEF | ITSN |
|  | Blackfly DNA Virus-16 SF02 377 | MK433230 | FLTYPQ | HHVHA | VEIR | GPSGIGKT | VFDEV | CTDS |
|  | Blackfly DNA Virus-17 SF02 1426 | MK433231 | CFTAFN | DHIQG | YCTD | GKTGVGKT | FLDEF | CSSK |
|  | Blackfly DNA Virus-18 SF02 66 | MK433232 | LITFPQ | PHWHA | YVAK | GQDLSDST | VFDDI | LINP |
|  | Blackfly DNA Virus-19 SF02 380 | MK433233 | SLTYAQ | LHFHC | YLKK | GPSRTGKT | IFDDF | LYNE |
| Circular DNA molecule | Blackfly DNA molecule 1 - rep | MK561604 | CFTINN | PHIQG | YCSK | GPTGTGKS | LIEDF | VTSN |
| Multi-component virus | Blackfly multicomponent virus 1 - rep | MK561605 | CFTINN | PHYQG | YCSK | GETGTGKS | IIEDF | VTSN |
|  | Blackfly multicomponent virus 2 - rep | MK561607 | CWTINN | PHLQG | YCSK | GDTGTGKS | LIEDF | VTSN |
